# Carrying out streamlined routine data analyses with reports for observational studies: introduction to a series of generic SAS ^® ^macros

**DOI:** 10.12688/f1000research.16866.2

**Published:** 2019-06-05

**Authors:** Yuan Liu, Dana C. Nickleach, Chao Zhang, Jeffrey M. Switchenko, Jeanne Kowalski

**Affiliations:** 1 Department of Biostatistics and Bioinformatics, Emory University, Atlanta, GA, 30322, USA; 2 Biostatistics and Bioinformatics Shared Resource, Winship Cancer Institute, Emory University, Atlanta, GA, 30322, USA; 3 Department of Oncology, University of Texas, Austin, Texas, USA

**Keywords:** SAS® macros, observational studies, streamlined data process, reporting, collaborative, Good-Research-Practice

## Abstract

For a typical medical research project based on observational data, sequential routine analyses are often essential to comprehend the data on hand and to draw valid conclusions.  However, generating reports in SAS ^® ^for routine analyses can be a time-consuming and tedious process, especially when dealing with large databases with a massive number of variables in an iterative and collaborative research environment. In this work, we present a general workflow of research based on an observational database and a series of SAS ^®^ macros that fits this framework, which covers a streamlined data analyses and produces journal-quality summary tables. The system is generic enough to fit a variety of research projects and enables researchers to build a highly organized and concise coding for quick updates as research evolves. The result reports promote communication in collaborations and will escort the research with ease and efficiency.

## Introduction

The increasing availability of large-scale medical registry databases (e.g. SEER ^1^, NCDB ^2^), health insurance claim databases (e.g. the US Food and Drug Administration’s Sentinel Initiative ^3^, MarketScan Research Database ^4^), electronic medical record databases (EMR), or secondary data from clinical trials provides opportunities for researchers and policymakers to address a variety of clinical practice questions and make informed decisions. A retrospective or observational study based on such data allows researchers to examine medical care in a real-life setting, and, if carefully done, generalize results to an extended population and clinical setting. With a large pool of patients, longer follow-up periods, and an affordable cost, such studies can address broader research questions with deeper insights. Such study designs also hold inherent limitations, such as selection bias (e.g., certain groups of patients are more likely to access a certain therapy) and confounding (e.g., the observed treatment effect might mix with the effects of other important prognostics factors that are imbalanced among treatment arms). It is believed that through thoughtful design, careful analysis, accurate interpretation, and transparent reporting, a sound scientific conclusion can be reached with minimized limitations ^5– 7^. However, even when well equipped with the concepts of good research practice ^8– 12^, a researcher holding a promising hypothesis with access to an excellent data source may face many challenges. They may include a lack of understanding of the full extent of the massive data and its feasibility to answer the study question(s); the complexity of the data on hand; the need for tediously repetitive and time-consuming data processing; lack of transparency in data processing and reporting; or miscommunication among collaborators with mixed levels of experience and expertise. The main motivation of this work is to illustrate generic research and analytic framework for studies based on an observational database, to emphasize the importance of routine data analysis, and to introduce a series of SAS ^®^ macros ^13^ designed to aid the journey of research with ease and efficiency.

In the following section, we illustrate how the proposed SAS ^®^ macros fit into the research and analytic framework seamlessly and assist in improving the overall research quality. In the case study, we exemplify the usage of the proposed macros by a real-life research project based on the NCDB with detailed result interpretations and discussion.

## Methods

### A general analytic workflow in observational studies

**Figure 1.**
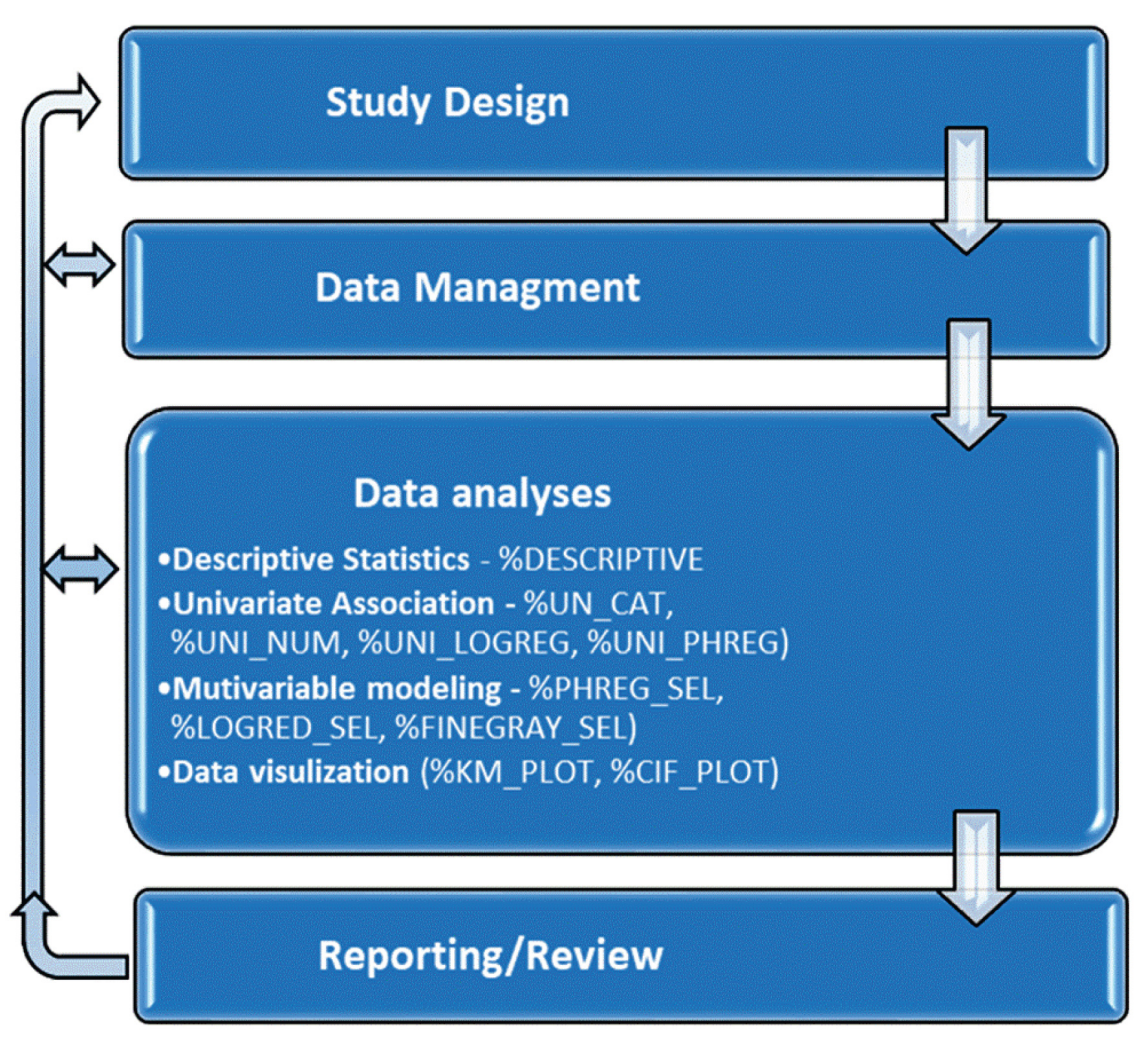
A general workflow of conducting research based on the observational database.

A general process of conducting research based on observational/retrospective studies involves a few general steps: study design, data management, data analyses, and reporting/review (shown in Figure 1), and each step interacts with each other as research is refined over time.

1. At the study design phase, the primary study goals or hypotheses need to be stated clearly with a suitable database along with proper definitions of study population, outcomes, cohorts, and covariates. A comprehensive literature review in the area further assists a better study design.
2. The data management step includes crafting the target study population by applying exclusion/inclusion criteria and preparing variables, such as creating derived variables, categorizing continuous variables, collapsing levels in categorical variables, handling missing values and outliers, etc. We recommend to assign an interpretable label and format to each variable for the best readable output table from the macros.
3. In the data analyses step, different layers of information about the data will be unfolded by sequential analytic steps to test ultimate study hypotheses. The routine data analyses, followed by more advanced analytic approaches, allow for building a pyramid of evidence to support hypotheses and hence strengthen study conclusions.
4. At the phase of reporting/review, we wrap up all results with interpretations in the context of the scientific background to evaluate the findings and draw conclusions with a statement of limitations. The process may involve reviews from an internal collaborative group or criticisms from journal reviews. There is some helpful guidance out there for reporting results from an observational study ^7, 11^.

The research itself should be dynamic and iterative as illustrated in Figure 1. Any issues, questions, criticisms, or new ideas that arise at a given step will redirect us to the previous steps and may lead to modifications of or additions to the original study design. The proposed SAS ^®^ macros will fit into this research framework seamlessly by covering routine data analyses in Step 3 more efficiently and enhancing reporting and communication in Step 4.

The routine data analyses serve as the foundational knowledge to support final claims in the research. The lack of correct perception or understanding of the importance of routine data analyses can lead researchers to rush into the final results without seeing the holes in the foundation, losing opportunities for improvement, and drawing biased conclusions. 1) For descriptive analyses, we get a chance to review the landscape of the entire study population and know the boundaries that apply to the conclusion. For example, if the study population is 99.9% Caucasian and 0.1% African American, the research findings may not be generalizable to the African American population or other racial groups. In addition, descriptive analyses help us to assess missing data, outliers, or possible data entry errors; and lead to additional data management. 2) For univariate associations or modeling, a natural association without any adjustment in the relationship between study cohorts and covariates or between outcome and cohorts/covariates will be demonstrated. We will learn of differences in patients’ characteristics among comparison cohorts, which may explain the differences we observe in the associations with the outcomes, especially when those characteristics are strong predictors of the outcomes. The phenomenon is referred to as selection bias or confounding, as commonly seen in observational studies. A confounder can be identified conceptually through existing knowledge or by examining the univariate associations as described here. 3) Both multivariable analyses and subgroup analyses are ways to deal with the confounding effects. Multivariable analyses are also known as main effects models which estimate an adjusted association with the outcome for each variable assuming all other variables are held constant in the model. The main effects model assumes the adjusted association with the outcome for each variable is uniformly constant across the levels of all other variables in the model. When such an assumption does not hold, subgroup analyses can be applied, known as interaction models, by which the association with the outcome is allowed to vary by the levels of a third variable (effect modifiers). Deeper technique details and strategies for multivariable modeling and variable selection, or more advanced approaches, such as propensity score methods, can be found in statistical textbooks or literatures ^14, 15^.

### SAS ^®^ macros

SAS ^®^ macros are powerful tools and have been used widely to build customized SAS ^®^ procedures to reduce repetitive coding. Many individual SAS ^®^ macros were created for reporting purposes ^16– 18^, but only suit very specific scenarios, and none of them cover a streamlined data analyses to truly serve the research generically and dynamically, as the proposed macros do.

### Implementation

The basic mechanism is similar in all proposed macros:

1. Process each variable in the list of interest or each analytic step one at a time, and continue such iteration until the last variable or step.
2. Inside each iteration, related SAS ^®^ exsiting procedures are carried out and the relevant and key information from the output is exported into SAS ^®^ datasets using ODS OUTPUT option.
3. Organize all information collected in step 2 into a concise and interpretable format by a series of DATA steps.
4. Report the final summary tables into a rich text file using PROC REPORT and ODS RTF option.

### Operation

All proposed SAS® macros work under SAS 9.3 and above (English version).

In Box 1, we list the proposed SAS® macros and their descriptions. They were developed in a highly collaborative and fast-paced National Cancer Institute-Designated Comprehensive Cancer Center research facility to help manage the standard workload involved in multiple, contemporaneous studies. The macros cover the report for the routine data analyses, such as descriptive, bivariate association and multivariable modeling for continuous, binary, ordinal, or time-to-event outcomes. Reporting for the univariate and multivariable generalized estimating equation (GEE) model for correlated data is also included ^19^. The benefit of using those macros is that they can handle a large number of variables at one time and create professional, consistent, and interpretable summary tables without copy-n-paste from the SAS output to the final report. Also, it results in readable and concise SAS code, and enables easier maintenance and revisions as the project evolves.

**Box 1. Selected SAS ^®^ macros and their description**

**Table:** 

Name	Function
***%DESCRIPTIVE***	a. Produce summary statistics (frequencies, percentages, means, medians, standard deviation, etc.) for all variables of interest in the database b. Provide a basic description of study population characteristics. c. Use to generate a data dictionary
***%UNI_CAT***	a. Examine univariate associations for multiple variables with a categorical outcome/endpoint or a categorical exploratory variable (e.g., study cohort). b. Report the parametric and non-parametric p-values (Pearson chi-square test and Fisher’s exact test for categorical covariates; and ANOVA and Kruskal- Wallis test for numeric covariates). c. Choose to report row or column percentage for contingency tables.
***%UNI_NUM***	a. Examine univariate associations for multiple variables with a numerical outcome/endpoint or a continuous exploratory variable (e.g., radiation dose). b. Report the parametric and non-parametric p-values (ANOVA and Kruskal-Wallis test for categorical covariates; and Pearson and Spearman correlation coefficient for numeric covariates).
***%KM_PLOT***	c. Produce Kaplan-Meier (KM) plots for time- to-event data. d. Generate summary statistic tables (e.g., median survival, accrual survival rate, and log-rank p-value). e. Produce multiple KM plots in one macro call for different combinations of data sets, strata variables, and outcomes.
***%UNI_PHREG***	a. Examine univariate associations between each variable of interest and a time-to- event outcome using Cox proportional hazards models. Optionally, competing risk analysis using the Fine & Gray model can be conducted. b. Report the frequency of categorical covariates, hazard ratio (HR) with 95% confidence interval (CI), log-rank p-value, and type-3 p-value. c. Allow for a counting process data format, setting the desired reference level of categorical covariates, proportional hazard assumption checks, and Firth’s bias correction.
***%PHREG_SEL***	a. Conduct backward selection on a Cox proportional hazards model at a user- specified significance level. b. Report the frequency of categorical covariates, HR with 95%CI, HR p-value and type-3 p-value. Report stratified HR when fitting an interaction model. c. Allow for a counting process data format, setting the desired reference level of categorical covariates.
***%UNI_LOGREG***	a. Examine univariate associations between multiple variables and a binary or ordinal outcome using a logistic regression model. b. Report the frequency of categorical covariates, odds ratios (OR) with 95%CI, OR p-value, and type-3 p-value. c. Allow for setting the desired reference level of categorical covariates, proportional odds assumption checks, and Firth’s bias correction.
***%LOGREG_SEL***	a. Conduct backward selection on a logistic regression model at a user-specified significance level. b. Report the frequency of categorical covariates, odds ratios (OR) with 95%CI, OR p-value, and type-3 p-value. Report stratified OR when fitting an interaction model. c. Allow for setting the desired reference level of categorical covariates.
***%FINEGRAY_SEL***	a. Conduct backward selection on a competing risk model. b. Report similarly as in %PHREG_SEL.
***%CIF_PLOT***	a. Produce a cumulative incidence plot using the proportional sub-distribution hazards model as proposed by Fine and Gray (1999). b. Generate time specific event rate with 95% CI and Fine and Gray p-value.
***%UNI_GENMOD***	a. Conduct univariate generalized regression analysis for each variable via PROC GENMOD, where normal, binomial, Poisson, or negative binomial distribution can be fitted. b. Can fit either GEE or non-GEE model. c. Report the parameter estimates with 95% CI, p-value and type 3 p-value. d. Allow for setting the desired reference level of categorical covariates
***%GENMOD_SEL***	a. Conduct backward selection on a generalized linear regression model using PROC GENMOD, where normal, binomial, Poisson, or negative binomial distribution can be fitted. b. Can fit either GEE or non-GEE model. c. Report the parameter estimates with 95% CI, p-value and type 3 p-value. d. Allow for setting the desired reference level of categorical covariates

## Use case

### Background

The traditional surgical approach for early-stage lung cancer is via a thoracotomy, and such open chest surgery is limited to certain eligible patients due to the increased risk of mortality, especially among elderly patients with multiple comorbidities. Over the past two decades, video-assisted thoracic surgery (VATS) has been increasingly used in clinics and provides excellent short-term advantages over thoracotomy, such as fewer complications, less pain, improved lung function, shorter recovery period and lower costs. On the other hand, incomplete lymph node evaluation with VATS or a high rate of the residual tumor may compromise the long-term efficacy of VATS vs. thoracotomy (open). The knowledge uncertainty still exists regarding the optimal surgical approach for early-stage lung cancer concerning long-term survival ^20^. The goal of this study is to compare the overall survival (OS) between two surgical approaches among the target eligible lung cancer patients.

The National Cancer Database (NCDB) ^2^, a joint project of the American Cancer Society and the Commission on Cancer of the American College of Surgeons, was queried for non-small cell lung cancer patients with clinical stage < T2N0M0 who underwent lobectomy in 2010-2013. After applying the exclusion/inclusion criteria, we identified 34,048 eligible patients. The study cohort is the surgical approach (open vs. minimally invasive). The clinical outcomes of interest were 30-day mortality after surgery (yes vs. no) and overall survival (OS): defined as months from the date of surgery to death or last follow up. Covariates considered included sex, Charlson-Deyo score (comorbidity), year of diagnosis, histology, AJCC clinic T stage, age at diagnosis and urban/rural 2003. For a detailed definition of these variables, users can refer to the online data dictionary ( http://ncdbpuf.facs.org/node/259?q=print-pdf-all).

In this case study, we show sequential analytical steps along with interpretations to probe into the study question by using the proposed SAS ^®^ macros. To make it easy to follow, we only include a short but relevant list of variables and cover routine data analyses. A comprehensive and complete manuscript for the study question is under preparation for a peer-reviewed publication.

### SAS ^®^ macros showcase – data analyses

Please note that these macros do not handle data cleaning or management directly. For the most interpretable results, it is highly recommended to properly format and label each variable before running these macros. All output tables are in Rich Text Format (RTF) and editable in Word.

***Descriptive Statistics***

The macro ***%DESCRIPTIVE*** was used to generate Table 1. The table provides an overview of the study population’s basic characteristics, in which the frequency and proportion are listed for categorical covariates and summary statistics (mean, median, min, max, standard deviation) for numerical covariates, along with the frequency of missing values. In the following SAS ^®^ code example, a few universal macro variables are implemented: **DATASET** for the data set name; **CLIST** for a list of categorical covariates separated by space; **NLIST** for a list of continuous covariates separated by spaces; **OUTPATH** for the file path to store the output RTF file; **FNAME** for the name of the RTF file; **DEBUG** to suppress deleting intermediate data sets for error checking by setting to T. Setting the **DICTIONARY** option to T creates two additional columns in the summary table, for the covariates’ SAS ^®^ names and unformatted values. These columns are useful for the programmer to connect the table with the dataset and code. In Table 1, we observe that 35.6% of the study population underwent a minimally invasive surgical approach, 45% were male, 80.9% resided in a metro area, and 85.4% had comorbidity score ≤1.

**Table 1.** Descriptive Statistics for All Variables.

Variable	Level	N (%) = 34048
Surgical Approach	Open	21911 (64.4)
	Minimally invasive	12137 (35.6)
Sex	Male	15324 (45.0)
	Female	18724 (55.0)
Urban/Rural 2003	Metro	26833 (80.9)
	Urban	5561 (16.8)
	Rural	756 (2.3)
	Missing	898
Charlson-Deyo Score	0	16637 (48.9)
	1	12440 (36.5)
	2+	4971 (14.6)
Year of Diagnosis	2010	8259 (24.3)
	2011	8175 (24.0)
	2012	8591 (25.2)
	2013	9023 (26.5)
Histology	Adenocarcinomas	21018 (61.7)
	Other or Unknown	4395 (12.9)
	Squamous cell carcinomas	8635 (25.4)
AJCC Clinical T	1	22688 (66.6)
	2	11360 (33.4)
Age at Diagnosis	Mean	67.02
	Median	68.00
	Minimum	40.00
	Maximum	90.00
	Std Dev	9.55
	Missing	0.00

************ SAS ^®^ code example for *%DESCRIPTIVE* **************************;

/* TIP 1: Create the macro variable &DIR to specify the location used to store all results. If you are conducting an updated analysis, you need to change this path, and all updated results will be saved to a new location without modifying it in every macro. */

%let dir = C:\Desired Location to Save All Results\;

/* TIP 2: Create the macro variables &cat_var and &num_var to store categorical and numerical variable lists outside macros, and reference them in all related macros. All related tables can be updated by changing those two macro variables.

/* TIP 3: Also note that the order of variables in CLIST is the same as the order in which they will appear in the final output table. Listing similar types of variables together, e.g., demographic variables, tumor characteristic variables, lab values, etc., will improve the readability of the results. */

/* Variable Label:

SEX - Sex

UR_CD_03 -- Urban/Rural 2003

CDCC_TOTAL -- Charlson Deyo score (comorbidity)

YEAR_OF_DIAGNOSIS -- Year of Diagnosis

HIS_CAT -- Histology

TNM_CLIN_T -- AJCC Clinical T stage

SURG_APP -- Surgical Approach

*/

%let cat_var = sex UR_CD_03 CDCC_TOTAL YEAR_OF_DIAGNOSIS HIS_CAT TNM_CLIN_T;

%let num_var = age ;

TITLE 'Table 1 Descriptive Statistics for All Variables';

% ***DESCRIPTIVE***(DATASET=anal,

     CLIST = surg_app &cat_var,

     NLIST = &num_var,

     OUTPATH= &dir,

     FNAME=Table **1** Descriptive Statistics for All Variables,

     DEBUG=F);

TITLE;

**************************************************************************;

***Univariate Association with a categorical outcome/exposure variable.***

We examined the univariate association between each covariate and surgical approach ( Table 2) using ***%UNI_CAT*** and 30-day mortality after surgery ( Table 3) using ***%UNI_LOGREG***. Each covariate was processed separately, but summarized all together in one table. ***%UNI_CAT*** is suitable to compare multiple covariates specified in **CLIST** and **NLIST** between two or more cohorts defined by a categorical variable ( **OUTCOME)** (see following SAS ^®^ code example). For each categorical covariate, frequencies from a contingency table are reported along with row percentages (Row%) or column percentages (Col%), which can be controlled by the **ROWPERCENT** option based on the desired interpretation. For each numeric covariate, summary statistics will be generated for each level of **OUTCOME**. The univariate associations can be tested by either parametric or non-parametric tests using the **NONPAR** option. The name of the tests will appear in the footnote of the table. Analyses can be performed using logistic regression models if the outcome/exposure variable is binary or ordinal using ***%UNI_LOGREG***. As shown in Table 3, the probability of the **EVENT** = ‘Yes’ was modeled and the odds ratio (OR) was reported with a 95% confidence interval (CI). The reference level of a categorical covariate in **CLIST** can be chosen to aid interpretation, which can be done by separating the CLIST variables by an asterisk ( *), and then adding “ **(DESC)”** or “ **(ref = “Reference level in formatted value”)”** after each desired variable name (see following code). If the outcome/explanatory variable is numeric, users can refer to SAS ^®^ macro ***%UNI_NUM***. In all report tables, a p-value < 0.05 will be shown in bold for easy visualization.

**Table 2.** Univariate association with study cohort.

			Surgical Approach		
Covariate	Statistics	Level	Open N=21911	Minimally invasive N=12137	Parametric P-value *
Sex	N (Col %)	Male	10128 (46.22)	5196 (42.81)	**<.001**
	N (Col %)	Female	11783 (53.78)	6941 (57.19)	
Urban/Rural 2003	N (Col %)	Metro	16863 (79.14)	9970 (84.18)	**<.001**
	N (Col %)	Urban	3894 (18.28)	1667 (14.08)	
	N (Col %)	Rural	550 (2.58)	206 (1.74)	
Charlson-Deyo Score	N (Col %)	0	10630 (48.51)	6007 (49.49)	**0.014**
	N (Col %)	1	7993 (36.48)	4447 (36.64)	
	N (Col %)	2+	3288 (15.01)	1683 (13.87)	
Year of Diagnosis	N (Col %)	2010	6107 (27.87)	2152 (17.73)	**<.001**
	N (Col %)	2011	5436 (24.81)	2739 (22.57)	
	N (Col %)	2012	5233 (23.88)	3358 (27.67)	
	N (Col %)	2013	5135 (23.44)	3888 (32.03)	
Histology	N (Col %)	Adenocarcinomas	13186 (60.18)	7832 (64.53)	**<.001**
	N (Col %)	Other or Unknown	2878 (13.13)	1517 (12.5)	
	N (Col %)	Squamous cell carcinomas	5847 (26.69)	2788 (22.97)	
AJCC Clinical T	N (Col %)	1	14132 (64.5)	8556 (70.5)	**<.001**
	N (Col %)	2	7779 (35.5)	3581 (29.5)	
Age at Diagnosis	N		21911	12137	0.520
	Mean		66.99	67.06	
	Median		68	68	
	Min		40	40	
	Max		90	90	
	Std Dev		9.54	9.57	

*The parametric p-value is calculated by ANOVA for numerical covariates and chi-square test for categorical covariates.

In Table 2, 42.8% of patients that had minimally invasive surgery were male, and 46.2% among patients that had open surgery. Compared to open surgical patients, minimally invasive surgical patients were more likely to reside in metro areas (84.2% vs. 79.1%), to be diagnosed in recent years, to have a histology of adenocarcinomas (64.5% vs. 60.2%), and to be at clinical T stage 1 (70.5% vs. 64.5%) (all p < 0.001). In Table 3, the factors associated with a higher risk of 30-day mortality after surgery were having open surgery (OR = 1.48), male gender (OR = 2.16), older age (OR = 1.06 for every 1-year increase), a comorbidity score larger than one (OR = 1.8 compared to score = 0), squamous cell carcinomas (OR = 2.49 compared to adenocarcinomas), and clinical T stage 2 (OR = 1.34) (all p < 0.001). By interpreting the information in Table 2 and Table 3 together, the univariate association between surgical type and 30-day mortality could be confounded by the effect from histology and clinical T stage. Open surgery was more likely to be conducted among patients who had squamous cell carcinomas and clinical T2, and these two factors were linked to a higher rate of 30-day mortality. The observed higher probability of 30-day mortality in open surgery patients might be partially due to the imbalanced distribution of histology and clinical T stage in the surgical cohorts. The common statistical approaches to control for confounding effects include multivariable analysis, subgroup analysis, and propensity score methods.

**Table 3.** Univariate association with 30-day mortality.

			30 Day Mortality=Yes		
Covariate	Level	N	Odds ratio (95% CI)	OR P-value	Type3 P-value
Surgical approach	Open	21911	1.48 (1.23-1.77)	**<.001**	**<.001**
	Minimally invasive	12137	-	-	
Sex	Male	15324	2.16 (1.83-2.55)	**<.001**	**<.001**
	Female	18724	-	-	
Urban/rural 2003	Rural	756	1.28 (0.77-2.12)	0.334	**0.013**
	Urban	5561	1.34 (1.10-1.64)	**0.004**	
	Metro	26833	-	-	
Charlson-Deyo score	2+	4971	1.80 (1.46-2.22)	**<.001**	**<.001**
	1	12440	1.09 (0.90-1.31)	0.373	
	0	16637	-	-	
Year of diagnosis	2010	8259	1.30 (1.05-1.62)	**0.018**	**0.031**
	2011	8175	1.00 (0.79-1.26)	0.990	
	2012	8591	0.99 (0.78-1.24)	0.915	
	2013	9023	-	-	
Histology	Other or Unknown	4395	1.13 (0.86-1.48)	0.393	**<.001**
	Squamous cell carcinomas	8635	2.49 (2.10-2.95)	**<.001**	
	Adenocarcinomas	21018	-	-	
AJCC clinical T	2	11360	1.34 (1.14-1.58)	**<.001**	**<.001**
	1	22688	-	-	
Age at diagnosis		34048	1.06 (1.05-1.07)	**<.001**	**<.001**

********** SAS ^®^ code example for *%UNI_CAT* and %UNI_LOGREG *************;

/* TIP 4: In the following example, the NONPAR option is turned off because it will take more time to compute Fisher’s exact test on a large sample. */

TITLE 'Table 2 Univariate Association with Study Cohort';

% ***UNI_CAT*** (

DATASET = anal,
OUTCOME = surg_app,
CLIST = &cat_var,
NLIST = &num_var,
NONPAR = F,
ROWPERCENT = F,
SPREAD = T,
OUTPATH = &dir,
FNAME =Table 2 Univariate Association with Study Cohort);

/* TIP 5: If you need to specify the reference level of a categorical variable, separate CLIST by “*” and add (DESC) or (ref = “Reference level in formatted value”) after each desired variable name. Otherwise, CLIST can be separated by spaces alone. */

%let cat_var_ref = SEX * UR_CD_03(DESC) * CDCC_TOTAL(DESC) * YEAR_OF_DIAGNOSIS * HIS_CAT(ref="Adenocarcinomas") * TNM_CLIN_T(DESC);

TITLE 'Table 3 Univariate Association with 30-day Mortality';

% ***UNI_LOGREG***(

DATASET = anal,
OUTCOME = PUF_30_DAY_MORT_CD,
EVENT = 'Yes',
CLIST = surg_app * &cat_var_ref,
NLIST = &num_var,
OUTPATH = &dir,
FNAME = Table **3** Univariate Association with **30**-day Mortality);

TITLE;

****************************************************************************;

***Univariate Association with a time-to-event outcome***

We examined the association of the study cohorts and each covariate with OS using ***%UNI_PHREG***, as shown in Table 4. Each covariate was individually fit in a proportional hazards model (PROC PHREG in SAS ^®^), and the hazard ratios (95% CI) and p-values were summarized in one table. In the following SAS ^®^ code example, the survival time variable is specified in **EVENT**, and the censoring indicator variable in **CENSOR**. Note that it requires that ‘1’ is used for the event and ‘0’ for the censored cases in the data ( **DATASET)**. Other options include **LOGRANK** to output log-rank p-value, **TYPE3** to output the Type3 p-values for categorical covariates, and **PHA** for proportional hazard assumption checks. Similar to ***%UNI_LOGREG***, one can set the reference level of a categorical covariate in **CLIST**. ***%UNI_PHREG*** can also handle time to event data in the counting process data form by using the **START** and **STOP** options, especially when modeling data with time-varying covariates or recurrent event data. It can also handle competing risk data by using the **EVENTCODE** option to activate the Fine and Gray’s model ^21^.

**Table 4.** Univariate association with overall survival (OS).

			Months (OS)		
Covariate	Level	N	Hazard ratio (95% CI)	HR P-value	Type3 P-value
Surgical approach	Open	21911	1.18 (1.13-1.24)	**<.001**	**<.001**
	Minimally invasive	12137	-	**-**	
Sex	Male	15324	1.68 (1.61-1.76)	**<.001**	**<.001**
	Female	18724	-	**-**	
Urban/rural 2003	Rural	756	1.23 (1.06-1.42)	**0.006**	**<.001**
	Urban	5561	1.19 (1.13-1.27)	**<.001**	
	Metro	26833	-	**-**	
Charlson-Deyo score	2+	4971	1.77 (1.67-1.89)	**<.001**	**<.001**
	1	12440	1.32 (1.26-1.39)	**<.001**	
	0	16637	-	**-**	
Year of diagnosis	2010	8259	1.00 (0.93-1.08)	0.900	0.922
	2011	8175	1.00 (0.93-1.08)	0.982	
	2012	8591	0.98 (0.91-1.06)	0.648	
	2013	9023	-	**-**	
Histology	Other or unknown	4395	1.05 (0.97-1.13)	0.211	**<.001**
	Squamous cell carcinomas	8635	1.64 (1.56-1.73)	**<.001**	
	Adenocarcinomas	21018	-	**-**	
AJCC clinical T	2	11360	1.63 (1.56-1.71)	**<.001**	**<.001**
	1	22688	-	**-**	
Age at diagnosis		34048	1.04 (1.03-1.04)	**<.001**	**<.001**

In the univariate analysis shown in Table 4, we see that patients who underwent open surgery had 18% more chance to die before those who underwent minimally invasive surgery (HR = 1.18; 95%CI = (1.13-1.24); p < 0.001). Also, residing in rural or urban areas, higher comorbidity scores, squamous cell carcinomas, clinical stage T2, and older age were all risk factors linked to worse overall survival in this study population. In combination with the results in Table 2, those prognostic factors except for age were also more likely to present in patients who underwent open surgery and should be controlled for as confounders in multivariable analyses.

************ SAS ^®^ code example for *%UNI_PHREG* and its options *****************;

TITLE 'Table 4 Univariate association with Overall Survival';

% ***UNI_PHREG*** (

  DATASET = anal,
  EVENT = os_surg,
  CENSOR = os_censor,
  CLIST = surg_app * &cat_var_ref,
  NLIST = &num_var,
  LOGRANK = F,
  TYPE3 = T,
  PHA = F,
  OUTPATH = &dir,
  FNAME = Table **4** Univariate association with Overall Survival);

TITLE;

*****************************************************************************;

***Multivariable model for the binary or time-to-event outcome***

We performed multivariable analysis with a logistic regression model for the 30-day mortality outcome using ***%LOGREG_SEL*** ( Table 5) and a Cox proportional hazards model for overall survival using ***%PHREG_SEL*** ( Table 6). The adjusted association of the surgical approaches with the two clinical outcomes was estimated after controlling for observed confounding variables. The odds ratio and hazard ratio were reported along with 95% CI and p-values. In both macros, a manual backward elimination procedure was implemented by dropping one variable at a time until all variables left satisfied a pre-specified alpha level (e.g., **SLSTAY = 0.1**). The selection process in the macros allows the sample size to adjust and always uses the maximum available sample as the number of variables in the model drops, which differs from the automatic selection procedure built into PROC LOGISTIC or PROC PHREG, where a complete and fixed data set is used throughout the selection procedure. In the following example code, **DSN** is for specifying the data set name, and **EVENT** in ***%LOGREG_SEL*** is to specify the event of interest, for which the probability will be modeled. **VAR** is for the list of all variables or terms in the initial model, separated by a space. **CVAR** is for the list of the categorical variables in **VAR** with the option to specify reference levels as shown in Table 4. **INC** = *k* is set to protect the first *k* variables in **VAR** from being eliminated, such as a primary exploratory variable and important confounding variables. In this case study, we want to keep surgical approach in the model as it is the study cohort, which was done by putting surg_app on the first position in **VAR** and setting **INC** = 1. Setting **CLNUM** = T outputs the frequency of categorical variables in the final model. The summary information of the selection procedure was presented in the footnote of the final report table. A separate macro for multivariable analysis of competing risk data is ***%FINEGRAY_SEL***. In reality, there are many approaches to build a multivariable model. If a user wants to customize the model building process, they can use these two macros for reporting purposes only by setting **VAR** = final model variables selected by other approaches and **INC** = total number of items in the final model.

**Table 5.** Multivariable logistic regression model for 30-day mortality.

		30 day mortality=Yes		
Covariate	Level	Odds ratio (95% CI)	OR P-value	Type3 P-value
Surgical approach	Open	1.41 (1.18-1.69)	**<.001**	**<.001**
	Minimally invasive	-	-	
Sex	Male	1.84 (1.55-2.18)	**<.001**	**<.001**
	Female	-	-	
Charlson-Deyo score	2+	1.44 (1.17-1.79)	**<.001**	**0.001**
	1	1.00 (0.83-1.20)	0.996	
	0	-	-	
Histology	Other or unknown	1.23 (0.93-1.61)	0.149	**<.001**
	Squamous cell carcinomas	1.94 (1.63-2.31)	**<.001**	
	Adenocarcinomas	-	-	
Age at diagnosis		1.06 (1.05-1.07)	**<.001**	**<.001**

* Number of observations in the original data set = 34048. Number of observations used = 34048. ** Backward selection with an alpha level of removal of 0.1 was used. The following variables were removed from the model: AJCC Clinical T, Urban/Rural 2003, and Year of Diagnosis.

**Table 6.** Multivariable cox proportional hazard model for overall survival.

			Months (OS)		
Covariate	Level	N	Hazard ratio (95% CI)	HR P-value	Type3 P-value
Surgical approach	Open	21307	1.12 (1.06-1.18)	**<.001**	**<.001**
	Minimally invasive	11843	-	-	
Sex	Male	14960	1.50 (1.43-1.58)	**<.001**	**<.001**
	Female	18190	-	-	
Urban/rural 2003	Rural	756	1.16 (1.00-1.34)	**0.049**	**<.001**
	Urban	5561	1.12 (1.06-1.19)	**<.001**	
	Metro	26833	-	-	
Charlson-Deyo score	2+	4866	1.59 (1.49-1.70)	**<.001**	**<.001**
	1	12153	1.26 (1.20-1.33)	**<.001**	
	0	16131	-	-	
Histology	Other or unknown	4287	1.11 (1.03-1.20)	**0.004**	**<.001**
	Squamous cell carcinomas	8424	1.28 (1.21-1.35)	**<.001**	
	Adenocarcinomas	20439	-	-	
AJCC clinical T	2	11077	1.49 (1.42-1.56)	**<.001**	**<.001**
	1	22073	-	-	
Age at diagnosis		33150	1.03 (1.03-1.04)	**<.001**	**<.001**

*Number of observations in the original data set = 34048. Number of observations used = 33150. **Backward selection with an alpha level of removal of .10 was used. The following variables were removed from the model: Year of Diagnosis.

In Table 5, the backward elimination drops clinical T stage, urban/rural and year of diagnosis from the 30-day mortality model at an alpha level of removal of 0.1, which means all variables in the final model have a p-value < 0.1. After controlling for the selected variables in the model, open surgery patients had a 41% higher odds to die within 30-days after surgery comparing to minimally invasive surgery patients (OR = 1.41; 95%CI = (1.18 - 1.69); p < 0.001). In Table 6, year of diagnosis was dropped from the final model to predict overall survival. Patients that underwent open surgery had 12% more chance to die before those that underwent minimally invasive surgery (HR = 1.12; 95% CI = (1.06-1.18); p < 0.001).

***Stratified Multivariable model***

When fitting the data into a main-effect model, as in Table 5 or Table 6, an imposed assumption is that the effect of treatment on outcomes is the same across all subgroups defined by the controlled variables. This assumption may or may not hold, and exploring and identifying subgroups that may benefit more from treatment can lead us to a deeper insight. Instead of splitting the dataset into smaller, separated data sets, fitting an interaction model on the entire data set is more appropriate ^22^. As shown in Table 7, we fit a multivariable model including an interaction term between surgical approaches and histology, still implementing the backward elimination procedure. In the related ***%PHREG_SEL*** code, the first three terms in **VAR** were protected from elimination by setting **INC** = 3, to keep the interaction of interest (surg_app*his_cat) in the model. The macro parameters **EFFECT** and **SLICEBY** allow users to specify the variables for the treatment effect and subgroups (the two variables have to be categorical variables for the macro to run correctly). Setting **SHORTREPORT** = T, only reports the hazard ratio (HR) and the p-value of surgical approach by histology subgroup, and if set to F, the HR for all other control variables in the model will be reported. This macro can only handle one interaction at a time but is useful as an initial exploration for the treatment effect in subgroups.

**Table 7.** Multivariable Cox model for overall survival (OS), stratified by histology.

		Months (OS)		
Covariate	Level	Hazard ratio (95% CI)	HR P-value	Type3 P-value
Comparisons stratified by histology	Surgical approach	-	-	0.069
Adenocarcinomas	Minimally invasive vs. Open	0.85 (0.79-0.91)	**<.001**	-
Other or unknown	Minimally invasive vs. open	0.95 (0.82-1.10)	0.511	-
Squamous cell carcinomas	Minimally invasive vs. open	0.96 (0.88-1.04)	0.321	-

*Number of observations in the original data set = 34048. Number of observations used = 33150. **Backward selection with an alpha level of removal of .10 was used. The following variables were removed from the model: Year of Diagnosis. ***The estimated stratified treatment effect was controlled by: AJCC Clinical T, Age at Diagnosis, Charlson- Deyo Score, Sex, Urban/Rural 2003.

In Table 7, detailed information about variable selection in the model building process is shown in the footnote. We see that, overall, minimally invasive surgery shows a protective effect on survival compared to open surgery, but such protection is more pronounced among adenocarcinoma patients (HR = 0.85; p < 0.001) than squamous cell carcinomas (HR = 0.96; p = 0.321). The p-value for interaction term is 0.069, and it tests the difference among the HR of 0.85, 0.95, and 0.96 for the three histology groups. If using the significance level of 0.05, the interaction p-value may confirm that the hazard ratio for the surgical approaches is the same across the histology groups. In the case of a significant interaction effect, researchers should report the interaction model and discuss the treatment effect in each subgroup.

******** SAS ^®^ code example for *%PHREG_SEL and %LOGREG_SEL* **************;

TITLE 'Table 5 Multivariable Logistic Regression Model for 30-day Mortality';

% ***LOGREG_SEL***(

  DSN = anal,
  OUTCOME = PUF_30_DAY_MORT_CD,
  EVENT = 'Yes',
  VAR = surg_app &cat_var &num_var,
  CVAR = surg_app*&cat_var_ref,
  INC = **1**,
  SLSTAY = **0.1**,
  CLNUM = F,
  OUTPATH = &dir,
  FILENAME = Table **5** Multivariable Logistic Regression Model for **30**-day Mortality);

TITLE 'Table **6** Multivariable Cox Proportional Hazard Model for Overall Survival';

% ***PHREG_SEL*** (

  DSN=anal,
  EVENT = os_surg,
  CENSOR = os_censor,
  VAR = surg_app &cat_var &num_var,
  CVAR = surg_app * &cat_var_ref ,
  INC = **1**,
  SLSTAY = **.10**,
  CLUNM=T,
  OUTPATH = &dir,
  FILENAME = Table 6 Multivariable Cox Proportional Hazard Model for Overall Survival);

TITLE 'Table 7 Multivariable Cox Model for OS Stratify by Histology';

% ***PHREG_SEL***(

  DSN=anal,
  EVENT = os_surg,
  CENSOR = os_censor,
  VAR = surg_app his_cat surg_app*his_cat &cat_var &num_var,
  CVAR = surg_app &cat_var ,
  INC = **3**,
  SLSTAY = **.10**,
  EFFECT = surg_app,
  SLICEBY = his_cat,
  SHORTREPORT = T,
  OUTPATH = &dir,
  FILENAME = Table **7** Multivariable Cox Model for OS Stratify by Histology);

TITLE;

*****************************************************************************;

***Kaplan–Meier analysis***

**Figure 2.**
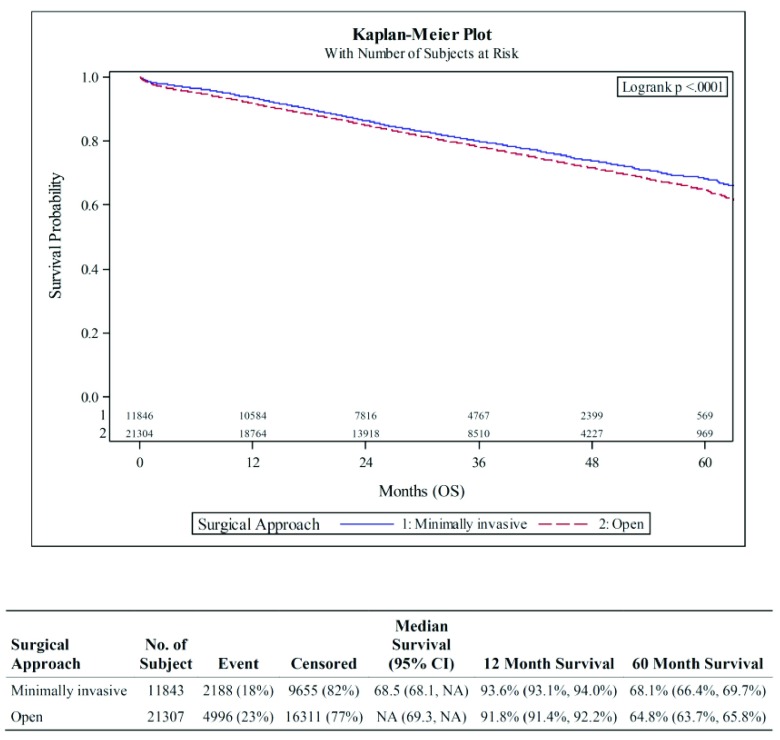
Kaplan–Meier plot of overall survival by surgical approach.

It is standard practice in many time-to-event studies to report Kaplan–Meier (KM) plots, median survival times, and accrual survival rates for time-to-event outcomes stratified by treatments, as a straightforward and intuitive way to assess the survival profile. ***%KM_PLOT*** was used to generate Figure 2. The KM plot for overall survival (specified in **EVENTS**, **CENSORS**) stratified by surgical approaches (specified in **GRPLIST**) was carried out with the key information of interest reported in a summary table. This macro has many options that allow the user to control the appearance of the plot ( **TITLE**, **XTICK**, **XMAX**, **NONCENSORED**, and **ATRISK**) and output the estimated accrual survival rate at pre-specified time points ( **TIMELIST**). This macro becomes handier when there are multiple datasets, several outcomes, or multiple variables of interest. Users can produce multiple KM plots in one macro call for all combinations of the parameter values by listing multiple values separated by spaces in **DSN**, **EVENTS**/ **CENSORS**, and **GRPLIST**.

In Figure 2, the two survival curves by surgical approaches do not reach the survival probability of 0.5, and hence the median survival time is not estimable (will show as NAs). The 60-month (5-year) survival rate is 68.5% in the minimally invasive surgery group, while 64.2% in the open surgery group. Overall, the log-rank p < 0.001 indicates a significantly improved overall survival in the minimally invasive surgery group over the open surgery group (note that the KM method is only univariate analysis). However, a gain of 4.3% improvement in 5-year survival rate may or may not be clinically significant, and a large sample size may easily lead to small p-values. In such cases, researchers may shed light on both the clinical relevance and statistical significance to interpret results. 

************ SAS ^®^ code example for %KM_PLOT *********** *******************;

% ***KM_PLOT***(DSN = anal,

  EVENTS = os_surg,
  CENSORS = os_censor,
  GRPLIST = surg_app,
  TITLE = "Figure 2 KM plot of Overall Survival by Surgical Approach",
  UNIT = Month, JOIN = T, PLOT = T, TABLE = T,
  TIMELIST = **12 60**, NONCENSORED = T,
  XTICK = ( **0 12 24 36 48 60**), XMAX = **60**, ATRISK = T,
  OUTPATH = &dir,
  FNAME = Figure **2** KM plot of Overall Survival by Surgical Approaches);

*****************************************************************************;

## Discussion

A natural course of research is generally iterative and conducted by a research team with mixed expertise and experience levels. The proposed sequential SAS ^®^ macros seamlessly fit into that type of research environment. They help process a massive amount of variables effortlessly, produce complete and interpretable summary information to support the research team to comprehend and better plan for the next step, and provide a highly-organized and concise coding interface that facilitates easy updates as research evolves.

Research based on observational or retrospective data needs extra effort in the study design and data management before data analysis, and careful interpretation afterward. The presented case study also illustrated a simple analytic workflow showing how to build an analytic project from the foundation, comprehend different layers of information jointly from the routine data analyses, and envision where you are and where to go. This work can serve as a nice tutorial for researchers to easily get their research off the ground.

The limitations include the requirements for researchers to be comfortable with the SAS ^®^ environment and have basic statistical training in data handling and interpretation. The proposed SAS ^®^ macros don’t handle study design or data management. A properly defined study population, cohort, outcomes, covariates, and sufficient literature review are critical before using our macros for the sake of research quality. Since only relevant information is summarized and reported, the macros don’t cover issues such as assumption check or goodness of fit about the fitted model at this time point. Including an experienced biostatistician in the research team would be beneficial.

These macros were first created in 2011 ^19^, and have been implemented in many projects to help turn out high-quality research, efficiently. They are still under active upgrade to meet new needs (e.g., ***%UNI_PHREG*** is in its 26 ^th^ version). At this time point, we are still lack of some desired features such as to allow users to decide the variable order in the final report, or to implement an automatic decision about the appropriateness of a parametric test or non-parametric test in ***%UNI_CAT*** or ***%UNI_NUM***, or to set the customized report template. However, those tasks along others are listed in our next round of upgrade and will become available soon. We welcome suggestions and comments that can help us improve.

## Data availability

Owing to data protection concerns, data used in the use case cannot be shared under the American College of Surgeons’ Commission on Cancer NCDB Participant Use File (PUF) Purpose and Terms of Agreement. For information on how to apply for access to NCDB PUF and who will be granted access to it, please visit: https://www.facs.org/quality-programs/cancer/ncdb/puf.

## Software availability

Source code available from: https://github.com/Emory-Yuan/BBISR-SAS-Macros/tree/V1.

Archived source code at time of publication: https://doi.org/10.5281/zenodo.2216377 ^13^.

License: MIT license.
