# Supplementary material for: Carrying out streamlined routine data analyses with reports for observational studies: introduction to a series of generic SAS ® macros
Source: F1000Res. 2019 Jun 5;7:1955. Originally published 2018 Dec 19. [Version 2] doi: 10.12688/f1000research.16866.2 (PMC6567291; doi:10.12688/f1000research.16866.2)
Supplement: Peer review history

# Peer review: Reviewer response for version 2

**DOI:** 10.5256/f1000research.21009.r49566

**Published:** 2019-06-13

**Authors:** Hong-Qiu Gu

**Affiliations:** 1 China National Clinical Research Center for Neurological Diseases, Beijing, China

I have no further concerns.

I have read this submission. I believe that I have an appropriate level of expertise to confirm that it is of an acceptable scientific standard.

# Peer review: Reviewer response for version 1

**DOI:** 10.5256/f1000research.18441.r46845

**Published:** 2019-04-26

**Authors:** Hong-Qiu Gu

**Affiliations:** 1 China National Clinical Research Center for Neurological Diseases, Beijing, China

Dr. Yuan Liu and colleagues had developed a series of generic SAS macros aiming to carry out streamlined routine data analyses and produce journal-quality summary tables. Although this work is fundamental, it’ll benefit statisticians a lot by avoiding time-consuming and tedious copy-and-paste processes. The idea of “streamlined routine data analyses” has been in my mind for years, and I do deem that all the available SAS macros in the SAS community are segmented for the whole data analyses process, thus, what we need is not more segmented SAS macros, but a set of SAS macros that can work seamlessly as an entire system. That’s why I developed the ‘%ggTable’ series of SAS macros and published ‘%ggBaseline’, the first macro of the ggTable series (Gu *et al.*, 2018 ^1^). Although other SAS macros of this series are still under preparation for a peer-reviewed publication, I’m glad to be a reviewer of this work and share some of my considerations that might help move this publication forward.

 Major considerations:

1. The title of this paper might be a little broad or exaggerated. Besides binary and time to event outcomes discussed in this article, multinomial and continuous variables can also be outcomes in observational studies. In addition, many statistical methods, such as propensity score-based methods, say stratifying, matching, and inverse probability weighting, are more and more popular nowadays in observational studies. Last, it’s common to use generalized estimating equation (GEE) models or mixed models to account for clustered data, since many centers are enrolled in observational studies. 

Minor considerations:

1. For %DESCRIPTIVE, %UNI_CAT and % UNI_NUM, the variables list is specified by CLIST and NLIST separately; this way has some drawbacks, such as 1) The order in the table will be grouped by variable type and always be categorical variables first; 2) Statistical tests and variable labels cannot be specified when calling the macros. However, this would not be a problem in %ggBaseline.
2. It would be better to report the mean with standard deviation (SD) in the same line as mean ± SD, the median with interquartile range (IQR) as median (IQR).
3. K-M plot with No. at risk is highly recommended, and the border of the legend should be removed as well.

It would be great if the authors can improve these SAS macros with the consideration of the above concerns. If there is no improvement, more discussions on these limitations and comparison to other available macros are needed in the discussion section.

I have read this submission. I believe that I have an appropriate level of expertise to confirm that it is of an acceptable scientific standard, however I have significant reservations, as outlined above.

## References

[1] GuHLiDLiuCRaoZ: %ggBaseline: a SAS macro for analyzing and reporting baseline characteristics automatically in medical research. *Annals of Translational Medicine*.2018;6(16) : 10.21037/atm.2018.08.13 10.21037/atm.2018.08.13 30364007PMC6186970

# Response

**Published:** 2019-04-30

**Authors:** Yuan Liu

**Affiliations:** Emory University, USA

We sincerely appreciate Dr. Gu’s vision and idea which agrees with our very initial motivation to create those macros (Dana et al, 2013, https://analytics.ncsu.edu/sesug/2013/PO-05.pdf), and her creation about %ggTable is elegant and useful to help us improve in the future.

For the major consideration, we do have several macros (%UNI_GENMOD, %GENMOD_SEL) that handle the reporting based on the generalized linear regression model (normal, binary, Poisson, negative binomial distribution) for either GEE (clustered data)or non-GEE (independent data) using PROC GENMOD procedure in SAS. In the revision, we added them into the Box 1 with a few more explanation in the context. Even the case study did not use them, they are all available in our final downloadable package, and the usage is similar as in other macros in the case study.

For the propensity score-based methods, we also created a series of macros for propensity score estimation, matching/weighting/stratification, balance check, visualization, etc. but since this paper was mainly focus on the routine data analyses, we decided to leave the PS related components and to develop a separate paper in the future.

We do appreciate that Dr. Gu pointed out some limitations and have included those limitations in the discussion section. However, we do actively upgrade those macros as we go to meet new needs. Since first created in 2011, some macros have reached their 20 ^th^ version. We would high appreciate any feedback and comments from the potential users and will improve with your help.

# Peer review: Reviewer response for version 1

**DOI:** 10.5256/f1000research.18441.r46844

**Published:** 2019-04-26

**Authors:** Margaret Stedman

**Affiliations:** 1 Department of Medicine, Division of Nephrology, Stanford University School of Medicine, Stanford, CA, USA

This article describes a series of SAS macros that automate a standard analysis of observational data. The macros include a descriptive analysis for table 1. An univariate analysis with the study outcome and study cohort and a multivariable or adjusted analysis. Outcome types include logistic regression, survival analysis, and competing risks.

   The authors do a good job describing the programs and giving examples of the output.  My main comment is that there are other macros available to create tables from SAS output and for someone who is adept at SAS the time savings may not be so great. However, it is nice to have them grouped together in a series. I wonder if the authors couldn't go a step further and make the user interface a little more user friendly (such as an ap or point and click) system? I think such system would be more useful to the general public that is not so adept at SAS.

I have read this submission. I believe that I have an appropriate level of expertise to confirm that it is of an acceptable scientific standard.

# Response

**Published:** 2019-04-30

**Authors:** Yuan Liu

**Affiliations:** Emory University, USA

We sincerely appreciate Dr. Stedman’s comments. Her suggestion about one more step further capture the trend of the current software development to enhance end-user experience, such as a web base application. I cannot agree more about her vision. Ideally, after most critical steps about study design and data management, data summary should just be one-click away. To relieve from manual data handling would allow to best use human brain power to focus on the data comprehension and critical thinking.
